# Patients with isolated REM-sleep behavior disorder have elevated levels of alpha-synuclein aggregates in stool

**DOI:** 10.1038/s41531-023-00458-4

**Published:** 2023-02-02

**Authors:** Anja Schaffrath, Sophia Schleyken, Aline Seger, Hannah Jergas, Pelin Özdüzenciler, Marlene Pils, Lara Blömeke, Anneliese Cousin, Johannes Willbold, Tuyen Bujnicki, Oliver Bannach, Gereon R. Fink, Dieter Willbold, Michael Sommerauer, Michael T. Barbe, Gültekin Tamgüney

**Affiliations:** 1 Institute of Biological Information Processing (Structural Biochemistry: IBI-7), Forschungszentrum Jülich, 52428 Jülich, Germany; 2 Department of Neurology, Faculty of Medicine and University Hospital Cologne, University of Cologne, 50923 Köln, Germany; 3 attyloid GmbH, 40225 Düsseldorf, Germany; 4 Institut für Physikalische Biologie, Heinrich-Heine-Universität Düsseldorf, 40225 Düsseldorf, Germany; 5 Cognitive Neuroscience, Institute of Neuroscience and Medicine (INM-3), Forschungszentrum Jülich, 52428 Jülich, Germany

**Keywords:** Diagnostic markers, Parkinson's disease

## Abstract

Misfolded and aggregated α-synuclein is a neuropathological hallmark of Parkinson’s disease (PD). Thus, α-synuclein aggregates are regarded as a biomarker for the development of diagnostic assays. Quantification of α-synuclein aggregates in body fluids is challenging, and requires highly sensitive and specific assays. Recent studies suggest that α-synuclein aggregates may be shed into stool. We used surface-based fluorescence intensity distribution analysis (sFIDA) to detect and quantify single particles of α-synuclein aggregates in stool of 94 PD patients, 72 isolated rapid eye movement sleep behavior disorder (iRBD) patients, and 51 healthy controls. We measured significantly elevated concentrations of α-synuclein aggregates in stool of iRBD patients versus those of controls (*p* = 0.024) or PD patients (*p* < 0.001). Our results show that α-synuclein aggregates are excreted in stool and can be measured using the sFIDA assay, which could support the diagnosis of prodromal synucleinopathies.

## Introduction

Parkinson’s disease (PD), dementia with Lewy bodies (DLB), and multiple system atrophy (MSA) are synucleinopathies characterized by the oligomerization and aggregation of α-synuclein into assemblies rich in cross-beta structure, which is a critical event in the pathogenesis of these diseases^1–6^. Because α-synuclein fibrils deposit in Lewy bodies and Lewy neurites in neurons in PD and DLB or in neuronal and glial cytoplasmic inclusions in MSA, small oligomeric intermediates of α-synuclein that are still soluble are thought to be the most toxic to neurons^7–9^. The diagnosis of synucleinopathies primarily relies on clinical assessment and brain imaging, which are either imprecise or costly, especially during early disease stages, highlighting the urgent need for a biomarker for early and reliable disease detection^10,11^. Also, for potential therapies to be most effective, detecting synucleinopathies at early stages may be advantageous when the burden of pathologic α-synuclein and neuronal loss are minimal. In the prodromal stage of synucleinopathies, non-motor symptoms, such as hyposmia and gastrointestinal dysfunction, can appear as early symptoms up to 20 years before cardinal motor symptoms manifest^10,12^. Also, most patients with isolated rapid eye movement (REM) sleep behavior disorder (iRBD), a parasomnia characterized by REM sleep without atonia and vivid dream enactment, convert to PD, DLB, or MSA within 10–20 years of diagnosis^13,14^. IRBD patients display many non-motor symptoms seen in PD patients including hyposmia, orthostatic hypotension, and gastrointestinal dysfunction^15^. Importantly, iRBD patients also accumulate pathologic α-synuclein assemblies in the central and peripheral nervous system^16,17^. Besides the nervous system and cerebrospinal fluid (CSF), α-synuclein aggregates have been detected in skin, olfactory mucosa, saliva, tears, urine, and blood of PD patients, which may all serve as potential sources for diagnosing synucleinopathies^18–26^. Histological findings also show that enteric neurons in the gastrointestinal submucosa of PD patients contain pathologic α-synuclein, even at prodromal stages^27–29^. Together with findings of pathological α-synuclein in the enteric nervous system (ENS) and the dorsal motor nucleus of the vagus nerve and the anterior olfactory bulb as the earliest lesion sites in PD, Braak and colleagues postulated the dual-hit hypothesis suggesting that α-synuclein pathology spreads from the olfactory bulb to the temporal lobes, and from the ENS via the vagus nerve to the CNS^30,31^. Further, several studies suggest that pathologic α-synuclein assemblies have prion-like properties, allowing them to multiply and propagate between neurons in the nervous system and to spread across large distances in the brain and body, including from the ENS to the CNS and vice versa^32–35^.

Earlier findings in transgenic TgM83^+/−^ mice expressing human α-synuclein demonstrated that α-synuclein fibrils used for an oral challenge could cross the mucosal barrier of the gastrointestinal tract after which they invaded the nervous system and triggered a synucleinopathy in these mice^36^. Here, we hypothesized that human pathologic α-synuclein assemblies released from, e.g., affected enteric neurons may traverse the mucosal lining of the gastrointestinal tract and be excreted in stool. This would involve a mechanism similar to the environmental shedding of prions in feces of deer and elk infected with chronic wasting disease or that of sheep and goats infected with scrapie^37,38^.

**Fig. 1:**
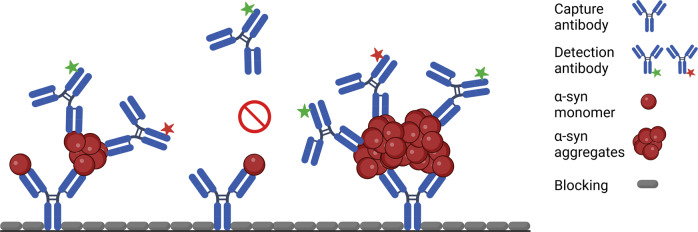
Schematic figure of the sFIDA assay principle. The glass surface of a microtiter plate is coated with a capture antibody (Syn211) against amino acid residues 121–125 of α-synuclein. The capture antibody binds both α-synuclein monomers and aggregates in the sample. The detection antibody (Syn211), a 1:1 mixture of this antibody labeled with fluorescent dye CF633 or CF488A, can only detect aggregates since it is directed against the same epitope as the capture antibody. The epitope on the monomer bound to the capture antibody is masked by the capture antibody and can, thus, not be bound by the detection antibody. The microtiter plate wells are imaged by two-channel confocal fluorescence microscopy to detect single particles. Only particles decorated with at least two different detection antibodies (colocalized signal) contribute to the sFIDA readout signal. The image was created with BioRender.com.

It remains challenging to specifically detect protein aggregates in body fluids as the expected concentrations are very low, requiring a high sensitivity that standard techniques such as ELISA do not provide^39^. Moreover, monomers are present in great excess, necessitating that assays have a high selectivity for oligomers. To overcome these challenges in detecting aggregated α-synuclein assemblies, we used the sFIDA (surface-based fluorescence intensity distribution analysis) assay, which uses antibodies targeting overlapping or identical epitopes, enabling it to specifically detect aggregates in the presence of monomers^40–43^. Here, the Syn211 antibody that recognizes amino acids 121–125 on α-synuclein is attached to a glass surface and captures α-synuclein species in stool homogenates^44^. Next, the captured α-synuclein species are detected with a blend of two Syn211 antibodies, each labeled with a different fluorescent dye, one green and one red (Fig. 1). The surface is then scanned by two-channel fluorescent confocal microscopy, yielding single particle sensitivity. Because the Syn211 antibody only recognizes a single linear epitope on α-synuclein, using the same Syn211 antibody to capture and detect α-synuclein species guarantees specific quantitation of only aggregated α-synuclein species. Monomers with a single linear epitope are captured but cannot be detected and are thus disregarded. Since the sFIDA assay in its current version does not differentiate between α-synuclein species from small oligomers to large fibrils, we refer to analytes measured in sFIDA as aggregates.

We recently used sFIDA to quantitate α-synuclein and tau aggregates in CSF of patients with PD, DLB, and other neurodegenerative diseases, showing that sFIDA is a reliable, highly sensitive, and robust assay for the diagnosis of neurodegenerative diseases and drug development^40^. The aim of this study was to detect and quantify α-synuclein aggregates in human stool and, more importantly, to demonstrate its possible use in a clinical setting and potentially for drug development. We adapted the sFIDA assay to quantify α-synuclein aggregates in stool samples of 217 subjects. We technically validated this assay by assessing parameters like the limit of detection (LOD), coefficient of variation (CV%), inter-assay correlation, and cross-reactivity to other aggregates.

## Results

### Descriptive analysis of the patient and control groups

We collected stool samples of PD (*n* = 94) and iRBD patients (*n* = 72) and healthy controls (HC) without past or present neurological disorder (*n* = 51) to determine if and how much α-synuclein aggregates they contain. Demographic and clinical information for these three cohorts on sex, age, education, disease duration, memory, motor, non-motor, olfactory, constipation, and further information are available in Table 1, and for individual patients and healthy controls in Supplementary Table 1. There was a gender bias towards men in the PD (69.1%) and more so in the iRBD (86.1%) cohort. In the control group, gender bias was slightly more towards women (60.8%). The age of PD (64.5 ± 9.9 years) and iRBD patients (66.3 ± 6.4 years) did not significantly differ, while healthy controls were on average 8–10 years younger (56.4 ± 17.1 years). We did not observe significant differences in the duration of education between the three groups. Disease duration in PD patients was 9.0 ± 5.8 years and in iRBD patients 7.4 ± 5.5 years. The cognitive performance (DemTect score) of healthy controls (15.9 ± 2.3) was significantly higher than that of PD (13.8 ± 3.3) and iRBD patients (14.8 ± 2.3, Supplementary Fig. 1a)^45^. PD patients (24.1 ± 14.9) scored significantly higher than iRBD patients (4.0 ± 2.7) on the Movement Disorder Society’s Unified Parkinson’s Disease Rating Scale Part III (MDS-UPDRS III)^46^. Equally, PD patients (30.6 ± 23.1) scored significantly higher on the Non-Motor Symptoms Scale (NMSS) for PD than iRBD patients (16.2 ± 18.0) or healthy controls (12.5 ± 14.4, Supplementary Fig. 1b)^47^. Also, PD patients (4.1 ± 4.0) were significantly more constipated than iRBD patients (2.8 ± 2.7) and healthy controls (2.5 ± 2.5), based on the Cleveland Clinic Constipation Scoring System (Supplementary Fig. 1c)^48^. PD patients (5.3 ± 2.6) also suffered more from hyposmia (Supplementary Fig. 1d), reflected in a significantly lower scent score, than iRBD patients (6.6 ± 2.7) or healthy controls (10.4 ± 1.7). In addition, PD patients (5.6 ± 2.2) scored significantly higher in the screening questionnaire for parkinsonism compared to iRBD patients (0.3 ± 0.8) and healthy controls (0.3 ± 0.8, Supplementary Fig. 1e)^49^. The results of the RBD screening questionnaire (RBDSQ, Supplementary Fig. 1f) also showed significant differences between iRBD (9.0 ± 2.7) and PD patients (4.9 ± 3.0) or healthy controls (1.7 ± 1.6), as well as between PD patients and healthy controls^50^. On average, PD patients scored 3.0 ± 0.9 on the Hoehn and Yahr scale and 40 ± 20% on the levodopa challenge test^51^.

**Table 1:** Demographic and clinical information on patients and controls that donated stool samples.

	PD	iRBD	HC	*p* (PD vs. HC)	*p* (PD vs. iRBD)	*p* (iRBD vs. HC)
Number	94	72	51	N/A	N/A	N/A
Female [number (percentage)]	29 (30.9)	10 (13.9)	31 (60.8)	0.001	n.s.	*p* < 0.001
Age [years ± SD]	64.5 ± 9.9	66.3 ± 6.4	56.4 ± 17.1	n.s.	n.s.	*p* < 0.05
Education [years ± SD]	15.4 ± 4.1	15.9 ± 4.0	16.0 ± 3.3	n.s.	n.s.	n.s.
Disease duration [years ± SD]	9.0 ± 5.8	7.4 ± 5.5	N/A	N/A	n.s.	N/A
DemTect [score ± SD]	13.8 ± 3.3	14.8 ± 2.3	15.9 ± 2.3	*p* < 0.001	n.s.	*p* < 0.05
MDS-UPDRS III [score ± SD]	24.1 ± 14.9	4.0 ± 2.7	N/A	N/A	*p* < 0.001	N/A
Non-Motor Symptoms Scale [score ± SD]	30.6 ± 23.1	16.2 ± 18.0	12.5 ± 14.4	*p* < 0.001	*p* < 0.001	n.s.
CCCSS [score ± SD]	4.1 ± 4.0	2.8 ± 2.7	2.5 ± 2.5	*p* < 0.05	*p* < 0.05	n.s.
Scent [score ± SD]	5.3 ± 2.6	6.6 ± 2.7	10.4 ± 1.7	*p* < 0.001	*p* < 0.05	*p* < 0.001
Screening questionnaire for parkinsonism [score ± SD]	5.6 ± 2.2	0.3 ± 0.8	0.3 ± 0.8	*p* < 0.001	*p* < 0.001	n.s.
IRBD screening questionnaire [score ± SD]	4.9 ± 3.0	9.0 ± 2.7	1.7 ± 1.6	*p* < 0.001	*p* < 0.001	*p* < 0.001
Hoehn and Yahr [score ± SD]	3.0 ± 0.9	N/A	N/A	N/A	N/A	N/A
Levodopa challenge test [% ± SD]	40 ± 20	N/A	N/A	N/A	N/A	N/A

*PD* Parkinson’s disease, *iRBD* isolated rapid eye movement sleep behavior disorder, *HC* healthy control, *SD* standard deviation, *MDS-UPDRS III* Movement Disorder Society’s Unified Parkinson’s Disease Rating Scale Part III, *CCCSS* Cleveland Clinic Constipation Scoring System, *N/A* not available, *n.s.* not significant.

### sFIDA measurements of α-synuclein silica nanoparticles (SiNaPs) and stool samples show a low intra-assay variance

**Fig. 2:**
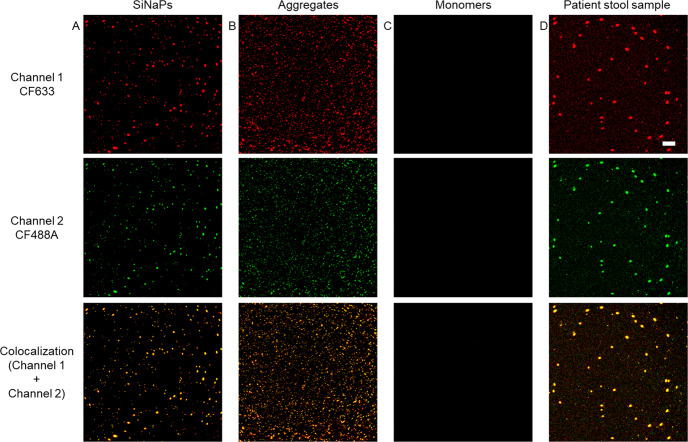
Fluorescence images of α-synuclein SiNaPs, synthetic α-synuclein aggregates, monomers, and a PD stool sample. Shown are exemplary fluorescence images taken in the red fluorescence channel (channel 1, CF633), green fluorescence channel (channel 2, CF488A), and the colocalized fluorescence signal of both channels of **a** 0.32 pM α-synuclein SiNaPs in buffer **b** 1581 pM synthetic α-synuclein aggregates in buffer (concentration based on the deployed monomer concentration), **c** 1000 pM α-synuclein monomers in buffer, and **d** a stool sample of a PD patient. To better visualize the 16-bit images, the contrast and minimum/maximum values were adjusted. Scale bar: 30 µm.

As a standard for signal calibration and LOD calculation, we used silica nanoparticles (SiNaPs), each carrying multiple monomers of α-synuclein^42^. Figure 2 shows exemplary images of the α-synuclein SiNaPs standard, synthetic α-synuclein aggregates, the buffer control, and a PD stool sample for the red fluorescence channel (channel one), the green fluorescence channel (channel two), and the colocalized signal of both channels. For analysis, only colocalized pixels (sFIDA readout) were utilized. Because of the high number of samples, the measurements had to be performed on nine 384-well microtiter plates. For all experiments, the mean intra-assay coefficient of variation (CV%) was calculated from the sFIDA readout of the four replicates of each sample to be 28.4% for the α-synuclein SiNaPs standard and 26.3% for the stool samples (Supplementary Table 2).

### Repeated sFIDA measurements of α-synuclein aggregates in stool samples show a low inter-assay variance

**Fig. 3:**
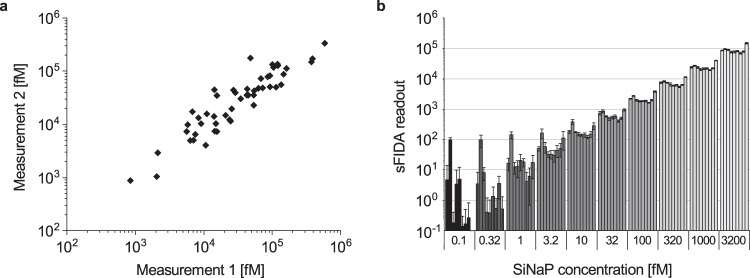
Repeated measurements of α-synuclein aggregates in stool and of α-synuclein SiNaPs yield highly replicable results. We tested the inter-assay variance of the sFIDA assay for α-synuclein aggregates. **a** Repeated measurements of 50 randomly selected stool samples by the same operator in the same laboratory on different days were highly reproducible with a Spearman coefficient of correlation of *r* = 0.916 (*p* = 0.2 × 10^−6^). **b** Nine independent measurements of a dilution series of the α-synuclein SiNaPs used for calibration of the stool sample data set also showed highly replicable results for all concentrations with a Spearman coefficient of correlation of *r* = 0.979 (*p* = 3.85 × 10^−62^). Error bars indicate standard deviation.

To assess the inter-assay variance between independent sFIDA assays, we repeatedly measured 50 randomly selected stool samples on two different days. Both measurements showed a significant correlation with a Spearman correlation coefficient of *r* = 0.916 (*p* = 0.2 × 10^−6^) (Fig. 3a). Next, we confirmed the reproducibility of the assay by repeatedly measuring the α-synuclein SiNaPs standard used for calibrating all stool samples in nine independent assays (Fig. 3b). The repeated measurements yielded highly reproducible results with a Spearman correlation coefficient of *r* = 0.979 (*p* = 3.85 × 10^−62^).

### sFIDA measurements of α-synuclein aggregates are highly selective

**Fig. 4:**
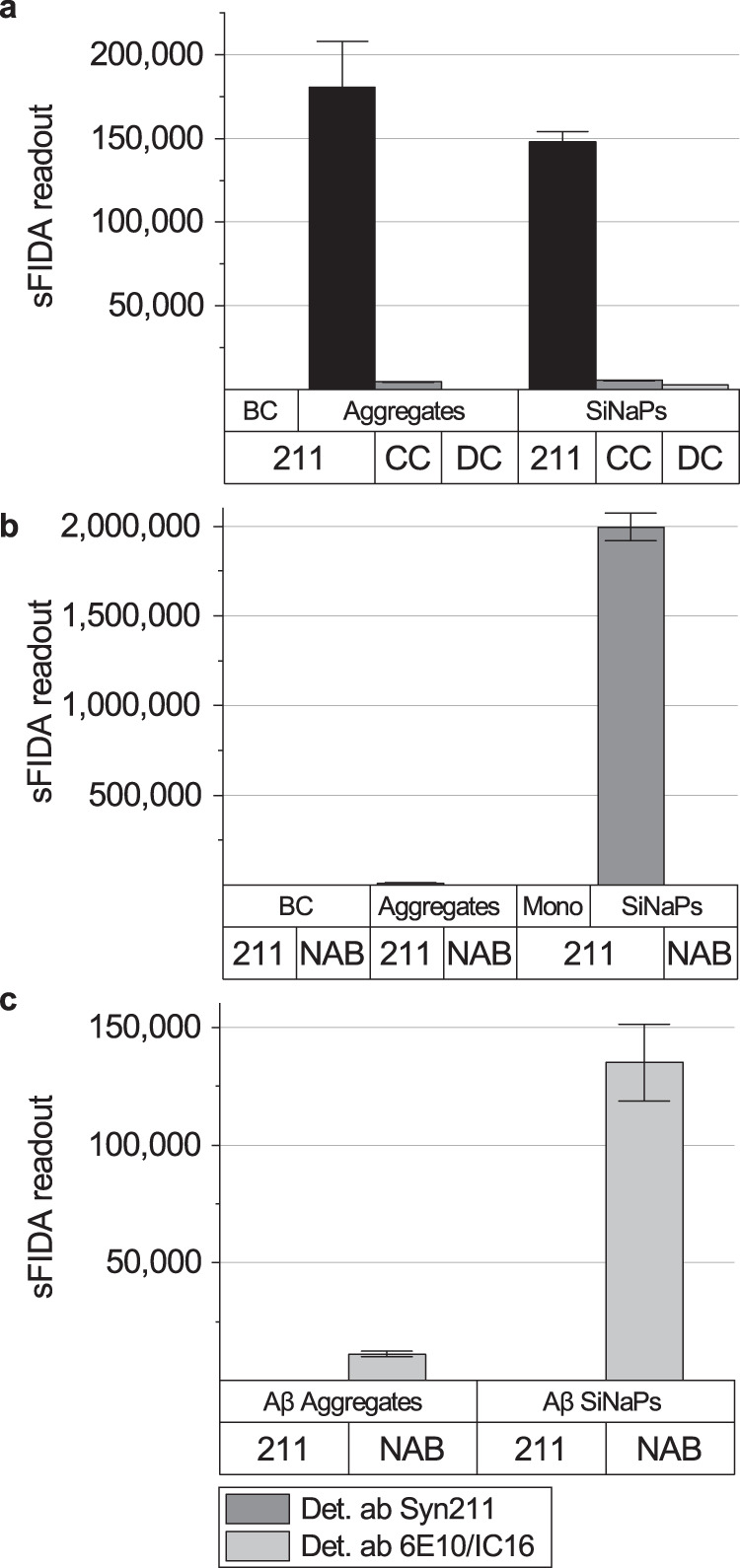
sFIDA with antibodies against α-synuclein specifically detects α-synuclein aggregates and α-synuclein SiNaPs but not α-synuclein monomers, Aβ aggregates, or Aβ SiNaPs. **a** When either the capture antibody or the detection antibodies were omitted (capture control (CC), detection control (DC)), the sFIDA readout for the CC was reduced by 96% for α-synuclein SiNaPs and 97% for α-synuclein aggregates of the respective readout with a capture antibody. For the DC, the sFIDA readout was reduced by 98% for α-synuclein SiNaPs, and entirely for α-synuclein aggregates (100%), where the sFIDA readout was indistinguishable from the buffer control (BC). **b** The sFIDA readout of α-synuclein SiNaPs and α-synuclein aggregates, when captured with the NAB228 antibody against beta-amyloid (Aβ), was reduced by almost 100% for α-synuclein SiNaPs and 98% for synthetic α-synuclein aggregates compared to the signal with Syn211 capture antibody. The sFIDA readout of α-synuclein monomers (Mono) was reduced to the level of the BC compared to the same concentration of monomers in aggregated α-synuclein. **c** Additionally, Aβ aggregates and Aβ SiNaPs were captured and detected with the Syn211 antibody against α-synuclein. As a positive control, the same Aβ aggregates and Aβ SiNaPs were captured with antibodies specific to Aβ (capture antibody: NAB228, detection antibodies: 6E10/IC16). The graph shows that the Syn211 antibody does not detect Aβ aggregates or Aβ SiNaPs. Error bars indicate standard deviation.

To further evaluate the sFIDA assay, we examined the ability of unspecific binding of synthetic α-synuclein aggregates or α-synuclein SiNaPs to the surface of the 384-well microtiter plate by comparing the readout in the presence and absence of a capture antibody. Relative to the readouts of α-synuclein aggregates and SiNaPs measured with a capture antibody, readouts without a capture antibody were reduced by 97% and 96%, respectively (Fig. 4a). We also measured samples in the absence of detection antibodies. This reduced the readout for synthetic α-synuclein aggregates fully (100%), which became indistinguishable from the generally low buffer control (BC) and for α-synuclein SiNaPs by 98%. Additionally, we tested the ability of the sFIDA assay to detect monomeric α-synuclein. As shown in Fig. 4b, the readout for α-synuclein monomers was as negligible as the BC readout and much lower than the readout of the positive control, which consisted of synthetic α-synuclein aggregates made from the same monomer concentration. Our results show that the potential presence of monomeric α-synuclein in stool, which most probably is degraded in this particular environment, did not influence the sFIDA readout. To determine the cross-reactivity of the sFIDA assay, we used the NAB228 antibody against amyloid-beta (Aβ) as a capture antibody and synthetic α-synuclein aggregates or SiNaPs as samples. This reduced the sFIDA readout for synthetic α-synuclein aggregates by 98% and that for α-synuclein SiNaPs by almost 100% (Fig. 4b).

At last, to determine the selectivity of the sFIDA assay, we measured synthetic Aβ aggregates and Aβ SiNaPs using the Syn211 antibody against α-synuclein for capture and detection. As a positive control for detecting Aβ aggregates and SiNaPs, we used NAB228 as a capture antibody and 6E10 and IC16 as detection antibodies. In contrast to this antibody combination specific for Aβ, antibodies specific for α-synuclein did not detect Aβ aggregates or Aβ SiNaPs (Fig. 4c).

### Immunodepletion removes α-synuclein aggregates in samples

**Fig. 5:**
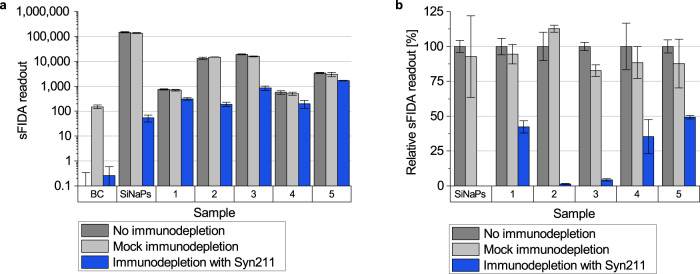
Immunodepletion of α-synuclein SiNaPs and α-synuclein aggregates in stool samples. **a** The α-synuclein SiNaPs standard and five stool samples were immunodepleted with magnetic beads coupled to Syn211 antibodies and, as a mock immunodepletion, without coupled antibodies. Immunodepletion entirely eliminated the sFIDA readout for α-synuclein SiNaPs (100%) and reduced that for α-synuclein aggregates in stool samples on average by 73%, ranging from 51% to 99% for different samples. Mock immunodepletion did not significantly affect the sFIDA readout with a reduction of about 7% for stool samples and α-synuclein SiNaPs. **b** The relative sFIDA readout [%] value represents the percent ratio of the sFIDA readouts of (mock) depleted to non-depleted samples.

To prove that sFIDA measurements are selective for α-synuclein aggregates, we performed an immunodepletion. Samples were incubated with dynabeads coupled to the Syn211 antibody specific for α-synuclein to remove α-synuclein aggregates in stool or α-synuclein SiNaPs. As a further control, samples were also incubated with blank dynabeads not coupled to antibodies for mock immunodepletions. After immunodepletion, supernatants were analyzed with sFIDA. The sFIDA readout for immunodepleted stool samples was on average decreased by 73%, and for immunodepleted α-synuclein SiNaPs by 100% (Fig. 5a, b). Mock immunodepletion of the same samples resulted in a reduction of only about 7% compared to readouts without immunodepletion (Fig. 5b). This minimal reduction could have been caused by proteolytic digestion or disassembly of aggregates, or unspecific adhesion of aggregates to beads during incubation at room temperature.

### sFIDA detects attomolar concentrations of α-synuclein SiNaPs

The sensitivity of the sFIDA assay was determined using the α-synuclein SiNaPs standard. To achieve this, a one-sided Mann-Whitney U test was applied to determine the lowest concentration significantly different from the buffer control for each SiNaPs standard to standardize the calibration range and determine the LOD. Based on these results, the lower limit of the calibration curve was set to 0.32 fM and the upper limit to 3200 fM. In all experiments, we observed a correlation between the sFIDA readout and the corresponding SiNaPs concentration with a Spearman correlation coefficient of *r* = 0.979 (*p* = 3.85 × 10^−62^). After calibration, the mean LOD was 0.3 fM for all experiments (for individual LOD values see Supplementary Table 3). For all measured samples, aggregate concentrations were above the LOD of the associated experiment (Supplementary Table 4).

### α-Synuclein aggregate concentrations are elevated in stool of iRBD patients

**Fig. 6:**
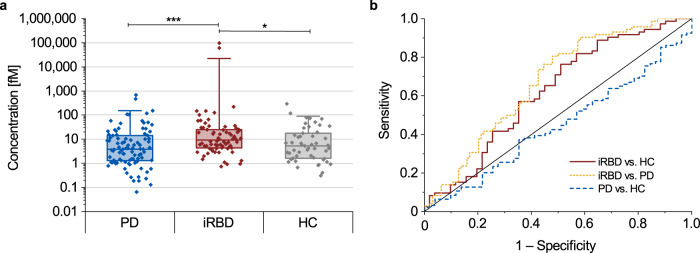
Calibrated sFIDA results and receiver operating characteristic (ROC) analyses for detecting α-synuclein aggregates in stool samples. Median concentrations of α-synuclein aggregates in stool samples of iRBD patients (9.2 fM) were significantly elevated compared to those of healthy controls (HC, 5.2 fM, *p* = 0.024) or PD patients (3.8 fM, *p* < 0.001). PD patients did not significantly differ from healthy controls. Box plots indicate the lower (Q1) and upper (Q3) quartiles as boxes. Horizontal lines within boxes indicate medians. Whiskers indicate 1.5 times the interquartile range (Q3–Q1) above Q3 and below Q1. Data falling outside this range are plotted as outliers. Significant differences between groups were calculated using the Kruskal–Wallis *H* test. ***p < 0.001, **0.001 ≤ *p* < 0.01, *0.01 ≤ *p* < 0.05. Error bars indicate standard deviation. **b** In the ROC analyses, discrimination of PD patients versus healthy controls showed a specificity of 96.1% and a sensitivity of 6.4%, with an area under the curve (AUC) of 0.447. In comparison, discrimination of iRBD patients versus healthy controls showed a specificity of 49.0% and a sensitivity of 76.4% with an AUC of 0.622 (see Table 2 for other specificity and sensitivity values and significances).

We finally used the sFIDA assay to measure α-synuclein aggregate concentrations in stool of PD and iRBD patients, and healthy controls. Using the α-synuclein SiNaPs standard, we calibrated the sFIDA readout of all samples and calculated α-synuclein aggregate concentrations (Supplementary Table 1). Statistical analyses were performed using non-parametric tests because none of the three groups showed a normal distribution (*p*-value < 0.05, Supplementary Table 5). The median concentration of α-synuclein aggregates in stool samples of iRBD patients (9.2 fM) was significantly elevated compared to those of the control group (5.2 fM, *p* = 0.024) as well as those of PD patients (3.8 fM, *p* < 0.001) when using the Kruskal–Wallis *H* test (Fig. 6a). Our findings for the iRBD cohort remained significant when the two highest outliers in this cohort were ignored or when adjusted for sex (*p* < 0.001). No difference was detected between PD patients and the control group. Receiver operating characteristic (ROC) curves (Fig. 6b) revealed a high sensitivity (>75%) of the sFIDA assay in detecting α-synuclein aggregates in stool of iRBD patients versus healthy controls or PD patients but a lower specificity (iRBD vs. PD: 52.1%; iRBD vs. HC: 49.0%; Table 2). In contrast, the sFIDA assay was highly specific (96.1%) but much less sensitive (6.4%) in detecting α-synuclein aggregates in stool samples of PD patients versus healthy controls. We also observed a weak positive correlation with a Spearman coefficient between the DemTect score and α-synuclein aggregate concentrations (*r* = 0.221, *p* < 0.05), and between the RBDSQ score and α-synuclein aggregate concentrations (*r* = 0.223, *p* < 0.05), but no other correlations in stool of PD patients, iRBD patients or healthy controls (Supplementary Table 6).

**Table 2:** Results of the ROC analyses for specificity, sensitivity, and area under the curve with the respective p-value for α-synuclein aggregates in stool.

	Specificity [%]	Sensitivity [%]	AUC	*p*
iRBD vs. HC	49.0	76.4	0.622	0.021
iRBD vs. PD	52.1	80.6	0.670	1.81 × 10^−4^
PD vs. HC	96.1	6.4	0.447	0.291

Parkinson’s disease (PD), isolated rapid eye movement sleep behavior disorder (iRBD), healthy control (HC), area under the curve (AUC).

## Discussion

We demonstrated that α-synuclein aggregates can be detected and quantified in stool, proving that not only α-synuclein aggregates are secreted from the body into the lumen of the gastrointestinal tract and excreted in stool, but also that the sFIDA assay can be applied to stool samples to reveal the existence of α-synuclein aggregates in a subgroup of humans. To investigate the origin and fate of α-synuclein aggregates in the gastrointestinal tract is essential in the future. Also, this provides opportunities to explore the concentration of α-synuclein aggregates in stool for the diagnosis of neurodegenerative diseases. By omitting the Syn211 capture or detection antibodies, changing the Syn211 capture antibody to one specific for Aβ, or changing the substrate to α-synuclein monomers, Aβ aggregates, or Aβ SiNaPs, we showed that the stool-adapted sFIDA assay measures α-synuclein aggregate and α-synuclein SiNaP concentrations very specifically and sensitively over a wide range of concentrations. We observed an overlap between patients and healthy controls, resulting in a specificity of 49.0% and a sensitivity of 76.4% for discriminating iRBD patients from healthy controls, and a specificity of 52.1% and a sensitivity of 80.6% for discriminating iRBD and PD patients. We were unable to discriminate PD patients from healthy controls. Our results are less specific but almost as sensitive as other studies aiming to discriminate α-synuclein aggregate concentrations in CSF samples of different synucleinopathies from healthy controls^40,52,53^. We are not aware of any other studies that have attempted to quantify α-synuclein aggregate concentrations in stool, making a comparison of our results with previous ones, e.g., in CSF is not straightforward. All measured stool samples showed concentrations above the determined LOD with values between 0.1 M and 100 pM, with the majority of the samples (87%) showing concentrations between 1 fM and 300 fM. Surprisingly, stool of iRBD patients showed significantly elevated levels of α-synuclein aggregates compared to healthy controls. In contrast to this, stool of PD patients not only showed significantly lower levels of α-synuclein aggregates than those of iRBD patients but also showed no difference to healthy controls (Fig. 6a). For CSF, it has previously been shown that α-synuclein aggregate concentrations are significantly elevated in PD and iRBD patients compared to healthy controls^24,40,52,54^. Further, several longitudinal studies in PD patients showed an increase of aggregated α-synuclein in CSF with disease progression^55,56^. One explanation of why we did not observe an increase of α-synuclein aggregate concentrations in stool of PD patients or a correlation with age or disease duration may be the recently proposed “brain-first” and “body-first” PD subtypes with different affection of the ENS, making it harder to observe a difference to healthy controls^57–59^. Still, separation of potential brain-first and body-first PD is most reliable in de novo PD using polysomnography to detect RBD. As we had mid-stage PD samples and only a questionnaire-based evaluation of RBD symptoms, we could not reliably separate our PD patient cohort in brain-first and body-first PD subtypes. Another explanation might be that levels of α-synuclein aggregates in stool of PD patients do not behave like those in CSF. More α-synuclein aggregates could be excreted in stool during prodromal and early phases of PD and less in advanced stages, similar to decreases in CSF concentrations of Aβ_42_ during the course of Alzheimer’s disease^60^. One could also speculate that the α-synuclein aggregates in stool during very early disease stages may not exclusively be shed into the gastrointestinal tract by affected submucosal neurons but also by a systemic disposal mechanism for α-synuclein aggregates via liver and bile that becomes less efficient during later disease stages. This may explain why α-synuclein aggregate concentrations in stool and CSF correlate well during early disease stages but less in later stages. To better understand the dynamics of α-synuclein aggregate shedding or release into stool, concentrations in longitudinally collected stool samples of iRBD and PD patients need to be assessed.

Our data show that iRBD patients with a high risk of developing synucleinopathies have significantly higher levels of α-synuclein aggregates in stool compared to healthy controls, stressing the value of the sFIDA assay in early disease detection^13^. Stool, next to CSF, skin, and nasal mucosa, where α-synuclein aggregates accumulate in iRBD patients, is now an additional valuable and easily accessible resource with a potential for diagnostic purposes^52,61–63^. Whether iRBD patients with high concentrations of α-synuclein aggregates in stool are more likely to convert faster to PD, DLB, or MSA, or are more likely to develop a particular type of synucleinopathy remains to be determined. Interestingly, we also measured high α-synuclein aggregate concentrations in stool of some control subjects. This could be either due to prodromal iRBD/PD pathology or other factors in the gastrointestinal tract that may cause a disease-independent aggregation of α-synuclein. According to the presumption mentioned above, it could also mean that these subjects still do have a very efficient disposal system for α-synuclein aggregates into the stool. It will be essential to assess whether these individuals are showing other prodromal signs of PD and to monitor them longitudinally.

This study has several limitations. Due to the specificity of the Syn211 antibody, which recognizes epitopes 121–125 of α-synuclein, this sFIDA assay cannot detect aggregates that are solely composed of fragments missing the C-terminus with this epitope, or have this epitope masked due to posttranslational nitration or phosphorylation of tyrosine 125^44,64^. However, the abundance of α-synuclein aggregates where all α-synuclein molecules within one aggregate may be missing this particular epitope or have it masked is likely to be very small. Furthermore, more recent studies suggest that tyrosine 125 of α-synuclein may not be phosphorylated in PD and DLB^65^. Also, sFIDA does not distinguish between α-synuclein aggregates that are seeding-incompetent or harmless to neurons (off-pathway) and those that may be harmful to neurons (on-pathway)^66,67^.

The sensitivity and specificity of this assay may be improved, for example, by combining measurements of aggregated α-synuclein with those of other potential biomarkers like phosphorylated α-synuclein, tau, glial fibrillary acidic protein, or neurofilament light chain that are expressed in the ENS and may be altered early in disease^68–71^. Another approach to improve this assay could be to extract and concentrate α-synuclein aggregates from stool samples, e.g., by immunoprecipitation, prior to their quantitation. Diet and the gut microbiome may likely affect the detection of α-synuclein aggregates in stool^72^. Stool has a complex matrix containing water, proteins, fatty acids, polysaccharides, bacterial biomass, undigested food residues, and bile that may inhibit antibody binding and mask α-synuclein aggregates from detection. Stool also contains proteases that may still be active during the preparation of the stool homogenates, which would mean that the measured values must be regarded as lower limits of the present α-synuclein aggregates. Also, using additional tissue and body fluid sources in combination with stool may further enhance the sensitivity and specificity of the sFIDA assay and improve diagnostic accuracy in discriminating between different patient cohorts and controls^52,61–63^. In summary, our results show that α-synuclein aggregates are excreted in stool and can be measured using the sFIDA assay, which may facilitate the diagnosis of prodromal synucleinopathies. This could be of value for developing therapies targeting early disease stages, which may result in better efficacy.

## Methods

### Synthesis of protein-coated silica nanoparticles (SiNaPs)

SiNaPs were used for calibration and to test selectivity^40^. The body of the SiNaPs was synthesized via the Stöber process and the surface aminated with (3-aminopropyl)triethoxysilane. Functionalized α-synuclein peptide fragments (amino acids 115–130, Peptides and Elephants, Henningsdorf, Germany) or Aβ peptide fragments (amino acids 1–15, Peptides and Elephants, Henningsdorf, Germany) containing a cysteamine at the C-terminus were cross-linked to the surface of aminated SiNaPs functionalized with maleimidohexanoic acid, which was previously activated with 1-ethyl-3(3-dimethylaminopropyl)carbodiimide and N-hydroxysuccinimide (Sigma Aldrich, St. Louis, USA) to enable reaction of the cysteamine with the maleimide group. For synthesis of protein-conjugated SiNaPs, 10% of the possible binding sites were functionalized by adding protein to dispersed SiNaPs. Protein-coupled SiNaPs were subjected to ultra-sonication for 10 min prior to each use. The same batches of α-synuclein or Aβ SiNaPs were used for all experiments. To serve as a calibration standard, the stock solution of α-synuclein SiNaPs was diluted in extraction buffer (EliA Calprotectin 2 Extraction Buffer (Phadia, Uppsala, Sweden) with 1× protease inhibitor cocktail (cOmplete EDTA-free Protease Inhibitor Cocktail, Roche, Basel, Switzerland)) in a half-logarithmic dilution series from 3200 to 0.1 fM.

### Characterization of SiNaPs

The size and shape of the aminated SiNaPs were analyzed using transmission electron microscopy^73^. For the aminated silica core, a mean particle size of 18.5 nm was determined. Inductively coupled plasma–mass spectrometry (ICP-MS) was used to determine the silicon concentration. The molar concentration of the SiNaPs was calculated using the silicon concentration and the known size, density, and shape^40^.

### Labeling of antibodies

Fluorescent antibodies were used for microscopy-based detection of aggregates. The mouse monoclonal anti-α-synuclein Syn211 antibody (Santa Cruz Biotechnology, Inc., Dallas, USA) was labeled with CF633 and CF488A (Biotium, Freemont, USA) and the anti-Aβ IC16 (Heinrich-Heine University, Düsseldorf, Germany) antibody was labeled with CF633 (Biotium, Freemont, USA)^40,44^. The labeling process was performed according to the manufacturer’s protocol. The dyes with activated succinimidyl esters were mixed with the antibody in carbonate buffer to react covalently with the amines of the antibody. The labeled antibody was purified using a polyacrylamide bead suspension (Bio-Gel P 30 Gel, Bio-Rad Laboratories, Inc., Hercules, USA). The concentration and degree of labeling were determined according to the manufacturer’s protocol.

### Patients and healthy controls

This study was approved by the Ethics Commission of the Faculty of Medicine of the University of Cologne (19–1644). PD and iRBD patients and healthy controls were enrolled at the Department of Neurology of the University Hospital Cologne. Informed consent was obtained from all human participants. All enrolled subjects completed a basic characteristics questionnaire providing information on items, such as age, sex, handedness, years of education, and medical history. All subjects completed the validated German version of the screening questionnaire for parkinsonism originally developed by Tanner and colleagues^49^, the REM sleep behavior disorder screening questionnaire (RBDSQ)^50^, for which a score of six was determined as a cut-off value for detecting iRBD^74^, the Cleveland Clinics Constipation Scoring System (CCCSS)^48^, the Ocular Surface Disease Index (OSDI)^75^, the revised Beck Depression Inventory (BDI-II)^76^, the Memory Assessment Clinic Questionnaire (MAC-Q)^77^, and the Functional Activities Questionnaire (FAQ)^78^.

PD patients were recruited from patient charts of our outpatient clinic or the ward when patients were admitted to the hospital. Patients were diagnosed with PD (*n* = 94) according to the Movement Disorder Society Clinical Diagnostic Criteria for Parkinson’s disease^79^. Clinical and demographic information, including disease duration, were acquired based on an investigator’s medical reports and careful anamnesis. In addition to the basic characteristics questionnaire, PD and iRBD patients were asked to complete the Non-Motor Symptoms Scale (NMSS) questionnaire for PD^46^, and the Movement Disorder Society’s (MDS) Unified Parkinson’s Disease Rating Scale Part II (MDS-UPDRS II)^46^. Cognitive performance was assessed using DemTect^45^. Motor function was assessed with the MDS-UPDRS Part III^46^. The Hoehn and Yahr scale^51^, and MDS-UPDRS Parts I and IV were rated by an investigator for each patient^46^. A smell test was performed for each subject according to a standardized procedure. Some but not all PD patients received a DaTSCAN and a levodopa challenge test (LCT) outside of this study.

IRBD patients were part of an ongoing study (*n* = 72) recruiting a local prodromal PD cohort in Cologne through newspaper advertisements from the community. Following a structured telephone interview, subjects with a high likelihood of having iRBD were invited to undergo polysomnography (PSG). We used a mobile SOMNOscreenTM plus device for overnight PSG^80^. Visual PSG scoring was performed on 30 s epochs as well as diagnosis of iRBD according to the American Academy of Sleep Medicine’s manual for the scoring of sleep and associated events, version 2.6^46^.

Healthy controls (*n* = 51) were recruited by advertisement, and, in many cases, the caregivers of the PD patients agreed to participate in the study. Healthy controls were free of any neurological disease. In addition to the basic characteristics questionnaire (see above), healthy controls completed the NMSS questionnaire for PD, the RBDSQ, and a smell test. Healthy controls with an RBDSQ score greater than five were excluded from the analysis^74^.

Stool samples from all subjects were collected between July 2020 and September 2021 using beakers with a screw cap and a removal spoon. Additionally, a paper towel stool catcher was used to avoid contamination. Stool samples were collected at various locations. If a stool sample was collected at home, in most cases, it was not older than two hours and stored in a plastic bag in a refrigerator until it was further processed. Pseudonymized samples were stored at −80 °C.

All patient-related data and samples were anonymized, and researchers were aware of the clinical data at the time of the sFIDA measurement. Data were subsequently tested for normal distribution (Shapiro Wilk, Lilliefors, Kolmogorov–Smirnov, Anderson Darling). A Kruskal–Wallis *H* test was used to identify differences between cohorts. If significant differences were determined, a pairwise comparison using a two-sided Mann–Whitney *U* test was conducted.

### Sample preparation

Stool samples were collected at various time points using beakers with a screw cap and a removal spoon. Stool samples were thawed at room temperature (RT) for 15 min and homogenized according to the manufacturer’s protocol of the EliA Stool Extraction Kit with minor changes (Phadia, Uppsala, Sweden). Using Simplix extraction tubes (Gaudlitz, Coburg, Germany), 17 mg stool were sampled and homogenized in 1300 µL cold extraction buffer through vortexing. The homogenate was centrifuged at 3000 × *g* for 5 min at 4 °C to pellet non-soluble components. The supernatant was aliquoted and stored at −80 °C until further use.

### Assay protocol

In the present study, we used SensoPlate Plus 384-well glass bottom microtiter plates (Greiner Bio-One l, Frickenhausen, Germany). The glass surface was incubated with the Syn211 antibody as capture at 7.5 µg/mL in 1x PBS buffer overnight at 4 °C. After washing five times with 80 µL TBS-T (TBS and 0.05% Tween20) and afterwards five times with TBS, the wells were blocked with 0.5% nonfat, dried milk powder in TBS-ProClin (TBS containing 0.03% ProClin) for 3 h at RT. The plate was washed again with TBS-T and TBS and 20 µL protein-conjugated SiNaPs diluted in extraction buffer, and 20 µL of the samples, thawed at RT for 15 min, were incubated for 1 h at RT. After washing five times with TBS, the wells were incubated for 1 h with the fluorescent detection antibodies Syn211-CF633 and Syn211-CF488A (each 0.625 µg/mL) in TBS-T containing 0.1% BSA, after which the wells were washed again with TBS. For measurement, the buffer in the wells was changed to TBS-ProClin. Each standard and sample were pipetted as four replicates. For detection of Aβ aggregates and Aβ SiNaPs, the NAB288 antibody (Sigma Aldrich, St. Louis, USA) was used at 2.5 µg/mL in PBS buffer for capture, and the fluorescent detection antibodies IC16-CF633 and 6E10-AF488 (Biolegend, San Diego, USA) each at 0.625 µg/mL for detection. All washing steps were carried out with an automated microplate washer (405 LS Microplate Washer, BioTek, VT, USA).

### Inter-assay measurements

For inter-assay measurements of the calibration curve and samples, the same assay was repeated by the same person with the same antibodies and materials. Repeatedly assayed samples were subjected to an additional freeze–thaw cycle.

### Immunodepletion

Immunodepletion was performed with magnetic Dynabeads Protein G (Invitrogen, Waltham, USA) to which the Syn211 antibody was bound via the Fc region according to the manufacturer’s protocol. Shortly, dynabeads were washed twice with PBS-T (PBS and 0.05% Tween20) and applied to a magnet to remove the supernatant. After washing, the beads were incubated with 0.1 mg/mL Syn211 antibody (to reach a target load of 8 µg/mL beads), shaking at 650 rpm for 100 min at RT. After two more washing steps (see above), the coated beads were diluted in PBS-T to 20 µg/mL dynabeads, respectively. To ensure that a signal loss did not result from a non-specific binding of sample components to dynabeads, we also performed a synthesis without the antibody. For immunodepletion and mock immunodepletion, we applied 0.5 mg of antibody-coated and non-coated dynabeads to a magnet and removed the supernatant. Then, 100 µL sample was added and incubated for 1 h at RT while rotating. After incubation, the dynabeads were pelleted with a magnet and the supernatant was transferred to a fresh tube. All samples were analyzed using sFIDA as described above.

### Image-data acquisition and analysis

Images were taken with an IRIS confocal fluorescence microscope (In Cell Analyzer 6500HS, GE Healthcare, Chicago, USA), using a 40× magnification objective with water immersion by two-channel imaging (channel one excitation: 642 nm, emission filter: 682.5/59 nm, exposure time: 3 s; channel two excitation: 488 nm, emission filter: 524.5/48 nm; exposure time: 4 s). A total of 16 images with 2040 × 2040 pixels each were acquired per well, covering 16% of the total well area. The acquired images were analyzed with the in-house developed sFIDAta software tool to ensure unbiased and automated image data analysis^40^. This included automatic detection and elimination of images containing artifacts and counting aggregate labeling pixels. In addition, a min-max filtering was applied, resulting in a removal of 10% of the images with the highest and lowest sFIDA readout per well. The sFIDA readout refers to the number of colocalized pixels of both channels. To reduce the background signal and compensate for fluctuations of the absolute fluorescence intensities, intensity cutoffs were determined for each experiment based on the buffer control. The cutoff is referred to as the gray-scale value of an image at which the rate of positive versus the total number of pixels in the buffer control equals a preset value^40,81^. By choosing a cutoff value, only pixels exceeding the cutoff value were considered for analysis. Additionally, we included a calibration in each measurement and converted the sFIDA readout to a SiNaP calibration-based concentration [fM] to ensure that differences in fluorescence intensity did not influence assay results. For inter-assay measurements, a cutoff value of 0.001% and, for the analysis of the stool samples, a cutoff value of 0.0003% were chosen.

### Statistics

#### General statistics

Statistical analyses were performed using SigmaPlot 11.0, IBM SPSS Statistics 28.0.1.1 (15), OriginPro 2020 SR1, or JASP 0.14.1. Mean and standard deviation were calculated based on the sFIDA readout of four replicates. The intra-assay variation is described by the coefficient of variation (CV%). To determine inter-assay variation, the Spearman coefficient of correlation was calculated for the replicate measurements of the samples.

#### Calibration

For calculating the calibration line, only concentrations of the SiNaPs standard were included that significantly differed from the blank control and were above the LOD. To this end, a one-sided Mann-Whitney U test was carried out. After calculating the calibration range for each experiment, a universal calibration range for all experiments was established. The LOD is defined based on Eq. (1):

1 $$LOD\;\left[ {pixel} \right] = sFIDA\;readout\;\left( {blank\;control} \right) + 3\sigma$$

For linear regression, the sFIDA readouts were weighted with 1/readout. The buffer control was used as a negative control for the calibration and for calculating the LOD.

#### ROC analysis

A receiver operating characteristic (ROC) analysis was performed on the acquired dataset. The optimal combination of sensitivity and specificity for a ROC curve was calculated with a maximized Youden’s index.

### Reporting summary

Further information on research design is available in the Nature Research Reporting Summary linked to this article.

## Supplementary information

Supplementary Material

Reporting Summary

## Data Availability

The authors confirm that the data supporting the findings of this study are available within the article and its supplementary materials.

## Code availability

For image data analysis, we used the sFIDAta software application, which can be made available upon request.
