# Supplementary material for: Patients with isolated REM-sleep behavior disorder have elevated levels of alpha-synuclein aggregates in stool
Source: NPJ Parkinsons Dis. 2023 Feb 2;9:14. doi: 10.1038/s41531-023-00458-4 (PMC9895074; doi:10.1038/s41531-023-00458-4)
Supplement: Supplementary file 1 — Supplementary Material [file 41531_2023_458_MOESM1_ESM.pdf]

Patients with isolated REM-sleep behavior disorder have elevated levels of alpha-synuclein aggregates in stool

Anja Schaffrath[1], Sophia Schleyken[2], Aline Seger[2], Hannah Jergas[2], Pelin Özdüzenciler[1], Marlene Pils[1,3], Lara Blömeke[1,3], Anneliese Cousin[1], Johannes Willbold[1], Tuyen Bujnicki[1], Oliver Bannach[1,3,4], Gereon R. Fink[2,5], Dieter Willbold[1,4], Michael Sommerauer[2,5], Michael T. Barbe[2], Gültekin Tamgüney[1,4]

SUPPLEMENTARY RESULTS

Supplementary Table 1 Demographic and clinical information and concentrations of α-synuclein aggregates in stool of individual subjects

Sample	*Diagnosis*	*Sex*	*Age at sampling [years]*	*Age at onset [years]*	*Disease duration [years]*	*Education [years]*	*CCCSS score*	*DemTect score*	*NMSS score*	*MDS-UDPRS III score*	*Scent score*	*Hoehn and Yahr score*	*Levodopa challenge test [% change]*	*DaTSCAN score*	*Screening questionnaire PD score*	*RBDSQ score*	*α-Synuclein aggregate concentration ± SD [fM]*
1	PD	Male	73	60	13	18	9	13	37	22	4	4	0.692	+	5	7	1.38 ± 0.22
2	PD	Male	68	54	14	10	10	13	17	27	6	3	0.333	N/A	6	10	0.88 ± 1.07
3	PD	Male	56	45	11	12	3	17	51	35	7	4	0.529	+	5	8	9.20 ± 2.85
4	PD	Male	71	61	10	17	1	11	67	65	8	5	0.239	+	9	6	104.73 ± 17.43
5	PD	Male	46	41	5	12	0	14	103	21	3	3	0.438	N/A	6	2	0.26 ± 0.25
6	PD	Male	72	57	15	19	6	14	54	28	5	5	0.469	N/A	4	5	0.98 ± 0.55
7	PD	Male	54	50	4	16	0	18	20	19	2	2	0.467	+	7	0	1.51 ± 0.88
8	PD	Female	71	49	22	12	4	9	67	20	9	3	0.481	N/A	9	4	5.58 ± 2.07
9	PD	Male	56	52	4	16	2	15	9	33	7	5	0.320	N/A	7	5	2.97 ± 0.73
10	PD	Male	62	50	12	20	0	18	9	10	2	2	0.462	+	8	3	4.28 ± 0.51
11	PD	Male	80	78	2	8	3	14	4	17	3	2	N/A	N/A	1	0	4.28 ± 1.87
12	PD	Female	78	63	15	11	18	17	48	76	2	5	0.431	N/A	6	4	108.40 ± 20.31
13	PD	Male	68	58	10	18	9	16	4	17	3	4	0.632	N/A	5	2	1.53 ± 0.73

14	PD	Female	80	66	14	13	11	13	34	25	7	4	0.528	+	7	1	0.40 ± 0.33
15	PD	Female	52	35	17	19	1	12	27	23	4	4	0.577	N/A	5	7	0.19 ± 0.08
16	PD	Female	74	60	14	17	9	10	21	50	2	5	0.323	N/A	9	6	4.28 ± 0.75
17	PD	Male	63	54	9	13	1	12	12	15	4	2	0.639	+	7	4	5.72 ± 0.89
18	PD	Male	74	62	12	12	3	11	21	8	0	2	0.320	N/A	2	3	10.52 ± 3.99
19	PD	Male	82	73	9	12	0	9	46	23	8	4	0.378	N/A	7	5	2.22 ± 0.39
20	PD	Female	50	39	11	14	2	11	16	22	7	2	0.459	+	5	2	1.14 ± 0.11
21	PD	Female	56	51	5	12	0	15	4	30	9	3	0.321	+	7	5	1.04 ± 0.48
22	PD	Female	60	51	9	17	3	17	16	16	7	2	0.423	+	7	2	0.60 ± 0.25
23	PD	Male	60	40	20	12	7	18	15	30	3	3	0.474	N/A	5	3	462.07 ± 46.48
24	PD	Male	57	39	18	15	7	12	42	41	6	3	0.400	+	5	8	7.56 ± 1.15
25	PD	Male	59	56	3	17	13	18	46	7	4	2	0.483	N/A	7	9	673.25 ± 19.52
26	PD	Female	58	58	0	20	2	18	52	16	1	2	N/A	+	0	11	2.52 ± 0.37
27	PD	Male	68	62	6	20	1	17	10	5	6	2	N/A	+	4	2	0.07 ± 0.06
28	PD	Male	76	74	2	13	1	11	21	35	6	3	N/A	N/A	6	8	2.49 ± 0.76
29	PD	Male	60	51	9	21	4	14	56	22	1	3	0.486	N/A	8	5	1.19 ± 0.30
30	PD	Male	59	54	5	19	4	18	28	33	6	3	N/A	+	7	12	3.87 ± 1.14
31	PD	Male	65	63	2	15	6	12	28	42	5	4	0.431	N/A	9	8	7.09 ± 0.54
32	PD	Male	41	40	1	22	2	18	7	11	10	2	N/A	+	2	3	6.89 ± 0.60
33	PD	Male	66	45	21	15	5	18	14	11	6	2	0.000	+	4	4	1.57 ± 0.52
34	PD	Female	60	50	10	13	12	13	27	19	6	3	0.429	+	8	7	3.51 ± 0.72

35	PD	Male	71	66	5	12	2	8	30	42	9	4	0.250	+	4	1	0.27 ± 0.09
36	PD	Male	58	54	4	15	2	11	42	10	8	3	0.692	N/A	7	6	1.30 ± 0.92
37	PD	Female	70	57	13	8	11	11	25	22	8	3	0.441	N/A	6	4	0.19 ± 0.03
38	PD	Male	55	41	14	9	12	7	30	50	6	4	0.447	N/A	8	6	1.32 ± 0.29
39	PD	Male	68	64	4	13	6	9	65	21	3	3	N/A	+	9	6	2.19 ± 0.35
40	PD	Male	78	71	7	18	7	10	10	19	7	3	N/A	+	8	12	45.51 ± 12.8
41	PD	Male	55	50	5	16	1	14	1	5	8	2	0.538	N/A	3	5	5.79 ± 2.57
42	PD	Female	49	44	5	15	5	18	13	9	8	3	0.000	N/A	7	3	1.47 ± 0.11
43	PD	Female	65	50	15	25	4	17	22	N/A	5	4	0.219	N/A	5	11	4.01 ± 0.43
44	PD	Male	41	37	4	20	3	14	17	18	6	3	N/A	+	6	1	2.78 ± 0.30
45	PD	Male	69	58	11	16	3	10	20	21	6	3	0.517	N/A	5	5	2.42 ± 0.28
46	PD	Male	63	53	10	13	8	14	21	13	10	2	0.313	+	7	8	4.61 ± 0.73
47	PD	Male	71	65	6	12	3	10	26	48	4	3	0.163	+	7	1	0.61 ± 0.38
48	PD	Female	78	64	14	20	6	18	23	30	4	3	0.286	N/A	7	2	28.42 ± 1.67
49	PD	Male	76	60	16	12	11	13	90	31	6	4	0.300	N/A	6	9	1.72 ± 0.22
50	PD	Male	55	55	0	30	0	17	2	5	7	2	N/A	+	1	3	10.82 ± 1.36
51	PD	Male	77	65	12	18	3	13	26	26	7	3	-0.023	N/A	4	8	57.33 ± 12.05
52	PD	Male	64	52	12	22	9	16	20	25	7	2	0.933	N/A	8	4	6.72 ± 1.13
53	PD	Male	83	73	10	22	8	18	36	54	5	4	N/A	N/A	6	7	28.19 ± 3.40
54	PD	Male	62	53	9	18	3	15	44	6	2	2	0.333	+	5	6	1.03 ± 1.01
55	PD	Female	70	63	7	14	0	12	18	13	6	3	0.368	N/A	8	4	3.82 ± 0.35

56	PD	Female	74	61	13	8	3	12	19	63	4	5	N/A	N/A	7	7	15.3 ± 0.73
57	PD	Female	77	50	27	13	1	18	67	30	2	4	0.567	N/A	9	7	48.39 ± 6.44
58	PD	Female	62	48	14	23	7	18	11	40	7	5	0.093	+	6	4	21.84 ± 2.24
59	PD	Female	75	49	26	16	2	9	36	25	2	3	N/A	N/A	5	4	2.97 ± 1.59
60	PD	Male	59	57	2	14	1	14	29	41	9	3	N/A	N/A	4	3	0.98 ± 0.48
61	PD	Male	68	58	10	14	0	18	24	24	0	2	0.609	N/A	7	4	16.89 ± 0.56
62	PD	Male	73	68	5	18	0	18	14	22	7	3	N/A	+	3	4	1.07 ± 0.07
63	PD	Male	57	49	8	15	1	14	13	10	9	2	0.545	N/A	3	2	10.23 ± 1.20
64	PD	Female	54	45	9	16	2	18	15	9	10	3	0.686	+	7	3	24.45 ± 9.80
65	PD	Female	57	45	12	12	2	18	122	26	7	2	0.720	N/A	8	7	27.74 ± 1.61
66	PD	Female	68	51	17	12	0	17	19	19	8	4	0.318	N/A	2	8	3.70 ± 0.30
67	PD	Female	64	57	7	20	8	13	59	21	8	3	0.629	N/A	8	11	0.23 ± 0.15
68	PD	Male	56	48	8	13	10	12	39	22	4	3	0.310	+	6	6	1.34 ± 0.68
69	PD	Female	74	73	1	17	2	15	15	16	8	3	N/A	+	6	9	119.51 ± 12.54
70	PD	Male	46	36	10	24	1	15	22	16	9	2	0.667	+	6	3	3.34 ± 0.58
71	PD	Female	69	56	13	11	7	15	51	28	4	3	0.415	N/A	7	6	0.43 ± 0.19
72	PD	Male	55	53	2	18	12	14	50	17	3	2	N/A	N/A	8	6	14.38 ± 9.20
73	PD	Male	78	60	18	17	2	12	36	59	3	5	N/A	N/A	7	5	14.16 ± 1.74
74	PD	Male	52	45	7	16	7	9	51	21	8	3	0.346	+	6	2	1.09 ± 0.48
75	PD	Female	57	55	2	15	2	18	12	6	6	3	0.733	+	1	1	49.21 ± 4.31
76	PD	Male	55	45	10	N/A	N/A	7	79	22	7	3	0.478	+	N/A	N/A	3.56 ± 1.25

77	PD	Male	60	57	3	10	0	12	55	36	2	3	N/A	+	1	1	7.97 ± 1.47
78	PD	Male	52	46	6	18	6	13	0	5	6	2	0.000	N/A	3	1	1.10 ± 0.57
79	PD	Male	51	40	11	14	7	8	10	31	4	4	0.485	+	5	5	10.30 ± 1.23
80	PD	Male	73	72	1	14	15	15	62	23	1	3	N/A	N/A	5	1	57.08 ± 5.86
81	PD	Male	66	55	11	12	0	14	51	13	7	2	N/A	+	4	2	4.46 ± 0.90
82	PD	Female	67	53	14	12	3	15	57	31	8	4	0.341	N/A	9	10	11.45 ± 1.64
83	PD	Male	72	66	6	13	1	15	13	12	5	3	N/A	N/A	7	9	20.57 ± 0.95
84	PD	Male	59	49	10	13	3	14	29	13	3	2	0.474	N/A	7	2	1.81 ± 0.66
85	PD	Male	74	68	6	8	2	9	6	42	6	N/A	0.543	N/A	4	1	0.13 ± 0.12
86	PD	Female	62	56	6	16	0	13	13	10	2	2	N/A	N/A	2	6	8.78 ± 0.89
87	PD	Male	71	64	7	19	0	18	18	29	3	3	N/A	N/A	5	1	0.60 ± 0.28
88	PD	Male	51	51	0	13	1	17	12	11	3	2	N/A	+	4	11	15.51 ± 2.65
89	PD	Female	57	56	1	14	3	13	24	6	8	2	N/A	+	5	4	1.34 ± 0.91
90	PD	Male	78	72	6	13	0	17	11	14	6	3	0.444	+	2	1	0.80 ± 0.41
91	PD	Male	62	59	3	22	1	14	13	7	7	2	N/A	+	2	3	40.25 ± 2.04
92	PD	Male	81	69	12	13	4	2	64	55	1	5	N/A	N/A	7	4	21.44 ± 1.33
93	PD	Male	64	63	1	18	0	13	12	4	2	2	N/A	+	3	3	151.19 ± 19.38
94	PD	Male	80	77	3	10	1	13	31	17	1	2	0.143	+	4	3	8.73 ± 1.92
95	iRBD	Male	56	N/A	N/A	21	2	17	6	1	11	N/A	N/A	N/A	0	6	10.01 ± 2.42
96	iRBD	Male	72	60	12	3	N/A	16	1	4	11	N/A	N/A	N/A	2	6	2.83 ± 1.15
97	iRBD	Male	61	N/A	N/A	16	N/A	14	4	5	11	N/A	N/A	N/A	0	6	10.99 ± 2.60

98	iRBD	Female	59	55	4	20	4	17	14	0	6	N/A	N/A	N/A	0	10	15.96 ± 1.49
99	iRBD	Male	78	N/A	N/A	14	N/A	17	15	4	8	N/A	N/A	N/A	0	10	6.04 ± 2.12
100	iRBD	Female	67	50	17	12	3	13	N/A	8	5	N/A	N/A	N/A	0	9	5.54 ± 1.44
101	iRBD	Male	63	63	0	16	2	17	3	5	10	N/A	N/A	N/A	0	8	0.74 ± 0.86
102	iRBD	Male	58	54	4	16	0	13	N/A	6	9	N/A	N/A	N/A	0	10	8.37 ± 1.95
103	iRBD	Female	64	61	3	21	0	15	N/A	3	3	N/A	N/A	N/A	0	7	4.34 ± 0.80
104	iRBD	Male	64	63	1	15	4	11	N/A	2	7	N/A	N/A	+	0	8	4.12 ± 0.80
105	iRBD	Male	70	60	10	N/A	0	14	12	5	10	N/A	N/A	N/A	0	12	5.11 ± 1.11
106	iRBD	Male	66	52	14	17	0	12	4	5	7	N/A	N/A	+	0	11	5.9 ± 0.68
107	iRBD	Male	73	71	2	N/A	N/A	12	2	4	6	N/A	N/A	N/A	N/A	N/A	24.42 ± 5.82
108	iRBD	Female	65	60	5	16	1	17	6	3	5	N/A	N/A	+	0	9	2.90 ± 0.83
109	iRBD	Male	72	62	10	11	2	14	N/A	5	9	N/A	N/A	+	0	N/A	7.87 ± 1.34
110	iRBD	Male	75	72	3	18	2	14	8	7	3	N/A	N/A	N/A	0	7	126.81 ± 19.12
111	iRBD	Male	69	64	5	14	0	17	9	4	8	N/A	N/A	+	0	12	1.10 ± 0.33
112	iRBD	Female	66	62	4	15	5	18	2	3	9	N/A	N/A	N/A	0	5	18.92 ± 2.02
113	iRBD	Male	59	56	3	19	3	9	22	5	7	N/A	N/A	+	0	9	32.97 ± 3.34
114	iRBD	Male	75	66	9	18	0	17	1	5	7	N/A	N/A	N/A	0	11	16.64 ± 2.23
115	iRBD	Male	55	40	15	21	0	11	0	8	12	N/A	N/A	N/A	0	5	6.29 ± 0.68
116	iRBD	Male	69	66	3	15	8	12	N/A	13	7	N/A	N/A	+	0	11	3.26 ± 0.44
117	iRBD	Male	54	49	5	12	5	12	48	3	8	N/A	N/A	+	5	13	19.17 ± 4.96
118	iRBD	Male	74	72	2	16	0	15	N/A	6	7	N/A	N/A	N/A	0	10	3.82 ± 0.69

119	iRBD	Male	66	61	5	15	3	18	21	7	6	N/A	N/A	N/A	0	10	0.93 ± 0.28
120	iRBD	Male	70	63	7	20	2	13	31	8	1	N/A	N/A	N/A	1	5	6.08 ± 0.29
121	iRBD	Male	67	50	17	13	0	17	2	10	7	N/A	N/A	N/A	0	10	4.15 ± 1.32
122	iRBD	Male	67	56	11	17	0	17	3	4	9	N/A	N/A	+	0	10	8.14 ± 1.95
123	iRBD	Male	62	47	15	31	4	14	N/A	5	9	N/A	N/A	+	1	9	7.49 ± 1.43
124	iRBD	Male	70	N/A	N/A	15	0	18	1	4	7	N/A	N/A	N/A	0	5	4.10 ± 0.48
125	iRBD	Female	63	62	1	19	12	14	N/A	0	4	N/A	N/A	N/A	0	8	4.64 ± 4.76
126	iRBD	Male	75	55	20	15	6	13	N/A	4	8	N/A	N/A	+	1	8	35.60 ± 2.14
127	iRBD	Male	67	61	6	16	5	13	23	1	3	N/A	N/A	N/A	0	11	17.04 ± 3.11
128	iRBD	Male	70	67	3	22	3	14	N/A	3	3	N/A	N/A	+	1	10	64.72 ± 3.37
129	iRBD	Male	74	72	2	12	2	11	N/A	5	5	N/A	N/A	N/A	0	7	5.35 ± 1.06
130	iRBD	Male	58	53	5	13	9	14	N/A	7	5	N/A	N/A	+	3	11	2.55 ± 0.47
131	iRBD	Female	77	66	11	16	1	15	4	5	10	N/A	N/A	N/A	0	12	15.40 ± 2.58
132	iRBD	Female	59	55	4	16	1	15	N/A	4	10	N/A	N/A	N/A	0	7	24.68 ± 1.03
133	iRBD	Male	68	60	8	18	2	18	N/A	4	7	N/A	N/A	N/A	0	9	4.34 ± 0.84
134	iRBD	Male	63	N/A	N/A	N/A	N/A	18	N/A	2	7	N/A	N/A	N/A	N/A	N/A	5.34 ± 0.62
135	iRBD	Male	64	62	2	12	5	14	102	5	10	N/A	N/A	N/A	0	13	2.89 ± 0.53
136	iRBD	Male	61	50	11	16	6	18	76	5	9	N/A	N/A	+	0	12	147.61 ± 8.84
137	iRBD	Male	64	60	4	N/A	N/A	11	9	2	5	N/A	N/A	N/A	N/A	N/A	96912.87 ± 14905.2
138	iRBD	Male	66	63	3	12	3	13	2	5	7	N/A	N/A	N/A	0	4	10.42 ± 1.18
139	iRBD	Male	63	62	1	15	2	14	9	0	3	N/A	N/A	N/A	0	5	46.81 ± 3.15

140	iRBD	Male	61	51	10	25	1	15	6	3	5	N/A	N/A	+	1	12	223.93 ± 16.15
141	iRBD	Male	58	54	4	15	2	17	6	1	6	N/A	N/A	+	0	3	5.30 ± 0.66
142	iRBD	Male	63	55	8	13	9	11	8	3	0	N/A	N/A	N/A	0	11	12.89 ± 0.74
143	iRBD	Male	59	47	12	22	2	14	5	2	9	N/A	N/A	N/A	0	11	1.99 ± 1.39
144	iRBD	Male	73	63	10	17	0	12	1	1	8	N/A	N/A	N/A	0	8	36.79 ± 4.54
145	iRBD	Male	69	54	15	18	8	18	14	0	6	N/A	N/A	+	0	10	30.49 ± 7.58
146	iRBD	Male	60	N/A	N/A	N/A	N/A	12	15	3	7	N/A	N/A	N/A	N/A	N/A	10.48 ± 4.54
147	iRBD	Male	63	53	10	17	0	18	25	3	2	N/A	N/A	N/A	0	7	1.46 ± 0.83
148	iRBD	Male	60	50	10	15	2	17	7	2	7	N/A	N/A	+	0	2	28.32 ± 4.72
149	iRBD	Male	74	71	3	19	1	18	18	9	9	N/A	N/A	+	0	12	6.67 ± 1.46
150	iRBD	Male	56	53	3	N/A	N/A	14	17	2	10	N/A	N/A	N/A	N/A	N/A	7.65 ± 0.55
151	iRBD	Male	72	58	14	8	1	17	12	1	5	N/A	N/A	N/A	0	12	59941.25 ± 16328
152	iRBD	Male	65	61	4	13	1	18	6	3	4	N/A	N/A	N/A	0	7	30.55 ± 1.42
153	iRBD	Male	74	68	6	12	6	15	13	2	6	N/A	N/A	N/A	0	10	19.34 ± 2.84
154	iRBD	Male	72	57	15	17	0	14	19	5	5	N/A	N/A	N/A	0	10	59.57 ± 4.69
155	iRBD	Male	67	65	2	11	4	12	11	1	3	N/A	N/A	+	0	6	24.92 ± 4.06
156	iRBD	Male	80	60	20	19	0	14	15	7	6	N/A	N/A	N/A	0	7	5.98 ± 2.15
157	iRBD	Male	68	63	5	18	6	15	40	2	4	N/A	N/A	+	0	12	18.98 ± 4.67
158	iRBD	Female	77	70	7	13	6	14	24	4	6	N/A	N/A	+	1	7	24.75 ± 1.54
159	iRBD	Male	74	69	5	17	1	14	16	2	0	N/A	N/A	N/A	0	11	100.09 ± 8.34
160	iRBD	Male	69	64	5	13	4	15	21	7	4	N/A	N/A	N/A	0	10	23.66 ± 1.97

161	iRBD	Male	60	59	1	11	3	16	29	1	4	N/A	N/A	N/A	1	13	0.94 ± 0.82
162	iRBD	Male	61	59	2	N/A	N/A	18	15	2	8	N/A	N/A	N/A	N/A	N/A	58.18 ± 5.88
163	iRBD	Male	57	38	19	17	0	15	38	3	7	N/A	N/A	N/A	0	12	10.91 ± 1.89
164	iRBD	Female	56	50	6	13	4	17	27	2	9	N/A	N/A	N/A	1	12	2.87 ± 0.87
165	iRBD	Male	68	48	20	N/A	N/A	11	40	N/A	7	N/A	N/A	N/A	N/A	N/A	1.29 ± 0.14
166	iRBD	Male	76	70	6	15	3	15	18	12	10	N/A	N/A	N/A	1	11	146.32 ± 6.96
167	HC	Male	18	N/A	N/A	12	3	15	N/A	N/A	10	N/A	N/A	N/A	0	2	4.29 ± 2.43
168	HC	Male	62	N/A	N/A	18	0	14	N/A	N/A	10	N/A	N/A	N/A	0	3	3.57 ± 2.43
169	HC	Male	70	N/A	N/A	16	7	10	N/A	N/A	11	N/A	N/A	N/A	0	0	33.19 ± 3.44
170	HC	Female	58	N/A	N/A	16	0	17	N/A	N/A	11	N/A	N/A	N/A	0	0	80.06 ± 24.21
171	HC	Female	74	N/A	N/A	11	4	12	N/A	N/A	10	N/A	N/A	N/A	1	1	73.96 ± 4.57
172	HC	Male	70	N/A	N/A	12	0	12	N/A	N/A	10	N/A	N/A	N/A	0	1	45.82 ± 2.48
173	HC	Female	55	N/A	N/A	15	1	18	N/A	N/A	11	N/A	N/A	N/A	0	1	2.03 ± 1.35
174	HC	Female	20	N/A	N/A	14	4	15	N/A	N/A	11	N/A	N/A	N/A	0	1	5.24 ± 0.46
175	HC	Female	20	N/A	N/A	14	5	16	N/A	N/A	10	N/A	N/A	N/A	0	3	6.01 ± 1.70
176	HC	Female	78	N/A	N/A	12	4	18	N/A	N/A	11	N/A	N/A	N/A	0	3	28.93 ± 4.84
177	HC	Female	66	N/A	N/A	14	0	17	N/A	N/A	11	N/A	N/A	N/A	0	3	13.36 ± 2.41
178	HC	Male	75	N/A	N/A	18	0	14	27	N/A	11	N/A	N/A	N/A	0	0	17.56 ± 1.81
179	HC	Male	74	N/A	N/A	10	2	13	8	N/A	10	N/A	N/A	N/A	0	0	1.74 ± 0.19
180	HC	Female	70	N/A	N/A	11	0	18	9	N/A	11	N/A	N/A	N/A	3	2	5.78 ± 1.81
181	HC	Female	63	N/A	N/A	20	4	18	1	N/A	11	N/A	N/A	N/A	0	2	4.15 ± 0.37

182	HC	Female	66	N/A	N/A	18	7	18	10	N/A	12	N/A	N/A	N/A	0	2	6.66 ± 0.43
183	HC	Male	34	N/A	N/A	16	7	17	1	N/A	11	N/A	N/A	N/A	0	1	51.35 ± 0.81
184	HC	Female	72	N/A	N/A	18	1	18	10	N/A	10	N/A	N/A	N/A	1	0	13.00 ± 2.49
185	HC	Male	77	N/A	N/A	23	5	15	0	N/A	11	N/A	N/A	N/A	0	2	6.84 ± 0.90
186	HC	Female	60	N/A	N/A	16	0	18	18	N/A	12	N/A	N/A	N/A	0	4	2.93 ± 1.01
187	HC	Female	64	N/A	N/A	13	2	18	N/A	N/A	N/A	N/A	N/A	N/A	1	2	19.37 ± 1.97
188	HC	Female	61	N/A	N/A	14	2	17	3	N/A	8	N/A	N/A	N/A	0	1	12.08 ± 1.34
189	HC	Male	36	N/A	N/A	20	0	18	4	N/A	11	N/A	N/A	N/A	0	1	0.84 ± 0.25
190	HC	Male	62	N/A	N/A	13	1	N/A	N/A	N/A	N/A	N/A	N/A	N/A	2	2	0.68 ± 0.22
191	HC	Female	60	N/A	N/A	20	0	18	18	N/A	2	N/A	N/A	N/A	0	0	0.86 ± 0.38
192	HC	Male	52	N/A	N/A	15	0	14	4	N/A	10	N/A	N/A	N/A	0	0	2.01 ± 0.23
193	HC	Female	66	N/A	N/A	19	2	15	1	N/A	10	N/A	N/A	N/A	0	0	38.59 ± 4.2
194	HC	Female	63	N/A	N/A	12	4	17	34	N/A	11	N/A	N/A	N/A	0	0	1.20 ± 0.10
195	HC	Male	59	N/A	N/A	16	1	N/A	11	N/A	11	N/A	N/A	N/A	0	2	0.30 ± 0.24
196	HC	Female	71	N/A	N/A	16	11	18	15	N/A	11	N/A	N/A	N/A	0	4	14.93 ± 4.15
197	HC	Female	70	N/A	N/A	12	0	17	16	N/A	6	N/A	N/A	N/A	0	4	70.07 ± 10.59
198	HC	Female	48	N/A	N/A	17	1	18	1	N/A	11	N/A	N/A	N/A	0	0	3.55 ± 1.91
199	HC	Male	65	N/A	N/A	18	2	18	4	N/A	11	N/A	N/A	N/A	0	2	0.40 ± 0.09
200	HC	Male	72	N/A	N/A	22	1	13	14	N/A	7	N/A	N/A	N/A	0	3	1.58 ± 0.47
201	HC	Female	50	N/A	N/A	15	2	18	18	N/A	11	N/A	N/A	N/A	0	5	1.42 ± 0.82
202	HC	Male	43	N/A	N/A	21	0	14	0	N/A	10	N/A	N/A	N/A	0	0	7.07 ± 1.02

203	HC	Male	57	N/A	N/A	22	0	15	3	N/A	11	N/A	N/A	N/A	0	1	1.56 ± 0.16
204	HC	Female	69	N/A	N/A	21	0	16	2	N/A	7	N/A	N/A	N/A	0	0	1.47 ± 0.14
205	HC	Female	48	N/A	N/A	13	3	17	3	N/A	12	N/A	N/A	N/A	0	0	10.77 ± 1.16
206	HC	Female	39	N/A	N/A	18	4	15	3	N/A	11	N/A	N/A	N/A	0	0	119.64 ± 11.56
207	HC	Female	42	N/A	N/A	17	3	18	21	N/A	11	N/A	N/A	N/A	0	0	3.28 ± 0.34
208	HC	Female	53	N/A	N/A	15	1	17	2	N/A	11	N/A	N/A	N/A	0	3	2.67 ± 0.43
209	HC	Female	24	N/A	N/A	18	6	17	18	N/A	11	N/A	N/A	N/A	2	1	1.15 ± 0.58
210	HC	Female	52	N/A	N/A	15	4	13	46	N/A	11	N/A	N/A	N/A	0	4	1.14 ± 1.24
211	HC	Female	20	N/A	N/A	14	8	18	10	N/A	12	N/A	N/A	N/A	0	5	5.54 ± 1.23
212	HC	Male	78	N/A	N/A	12	0	13	32	N/A	N/A	N/A	N/A	N/A	1	5	2.82 ± 0.96
213	HC	Male	26	N/A	N/A	13	0	18	2	N/A	11	N/A	N/A	N/A	0	0	4.18 ± 0.92
214	HC	Male	38	N/A	N/A	17	3	17	0	N/A	12	N/A	N/A	N/A	0	3	292.79 ± 91.38
215	HC	Female	81	N/A	N/A	13	4	10	71	N/A	12	N/A	N/A	N/A	4	5	35.53 ± 9.47
216	HC	Female	61	N/A	N/A	23	5	18	16	N/A	9	N/A	N/A	N/A	1	1	6.87 ± 1.11
217	HC	Male	64	N/A	N/A	18	3	13	20	N/A	11	N/A	N/A	N/A	0	2	0.93 ± 0.45

PD, Parkinson’s disease; iRBD, isolated rapid eye movement sleep behavior disorder; HC, healthy control; CCCSS, Cleveland Clinic Constipation Scoring System; NMSS, Non-Motor Symptoms Scale; MDS-UPDRS III, Movement Disorder Society’s Unified Parkinson's Disease Rating Scale Part III; RBDSQ, iRBD screening questionnaire; SD, standard deviation; N/A, not available

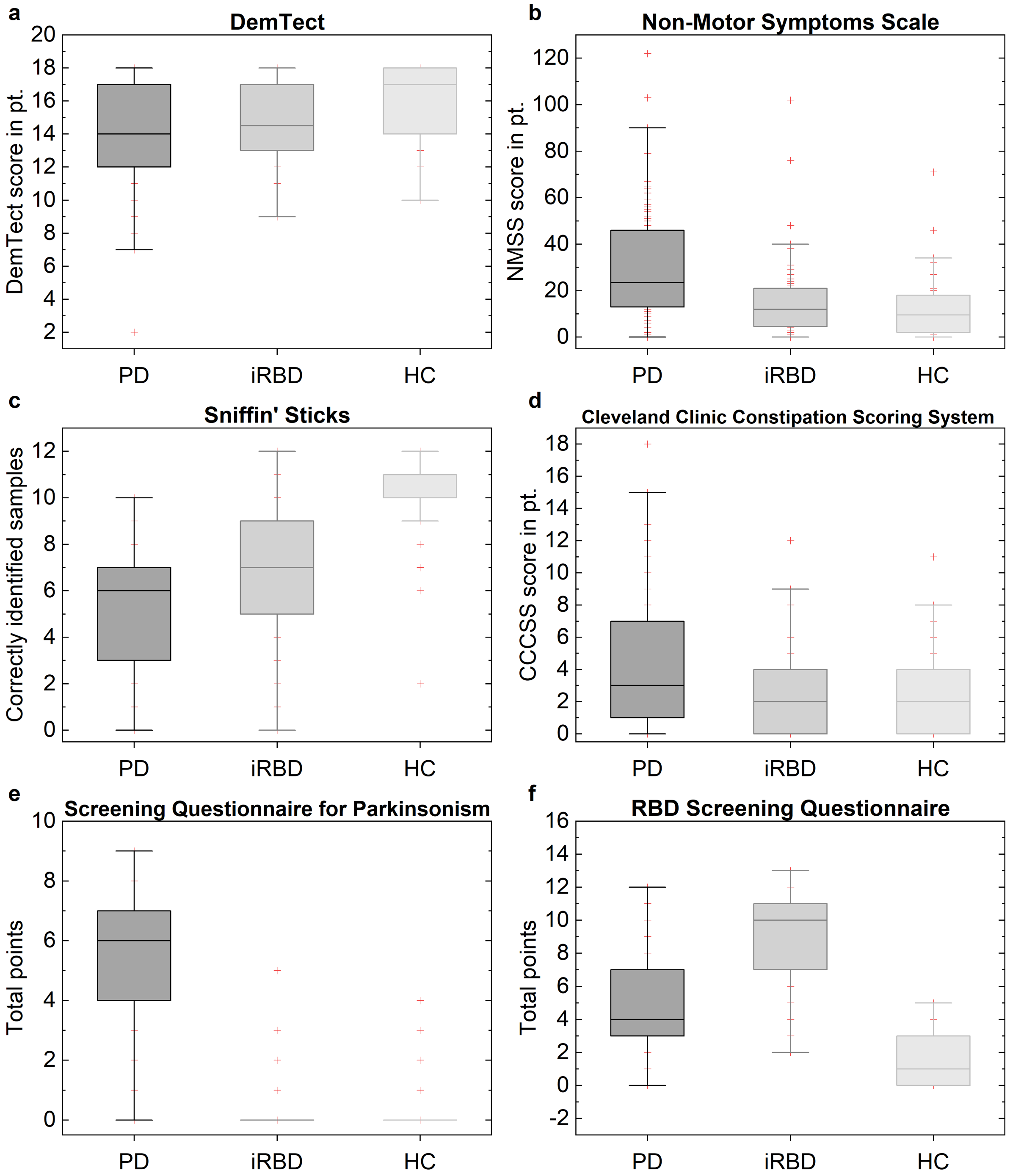

Supplementary Fig. 1 Results for PD and iRBD patients and healthy controls based on their performance in various tests and screening questionnaires.

a The DemTect score showed that the cognitive performance of healthy controls (15.9 ± 2.3) was significantly higher than that of PD (13.8 ± 3.3, $p < 0.001$) and iRBD patients (14.8 ± 2.3, $p = 0.020$). **b** Based on the Non-Motor Symptoms Scale (NMSS), the performance of PD patients was significantly worse than that of iRBD patients (30.6 ± 23.1 vs. 16.2 ± 18.0, $p < 0.001$) and healthy controls (12.5 ± 14.4, $p < 0.001$). **c** Also, the olfactory performance of PD patients (5.3 ± 2.6) was significantly worse ($p < 0.01$) than that of iRBD patients (6.6 ± 2.7) and healthy controls (10.4 ± 1.7), based on correctly

identified sniffin' sticks. **d** Based on the Cleveland Clinic Constipation Scoring System (CCCSS), PD patients (4.1 ± 4.0) were significantly more ($p < 0.05$) constipated than iRBD patients (2.8 ± 2.7) and healthy controls (2.5 ± 2.5). **e** PD patients (5.6 ± 2.2) scored significantly higher ($p < 0.001$) than iRBD patients (0.3 ± 0.8) and healthy controls (0.3 ± 0.8) on the Screening Questionnaire for Parkinsonism. **f** The RBD Screening Questionnaire (RBDSQ) revealed significant differences ($p < 0.001$) between iRBD (9.0 ± 2.7) and PD patients (4.9 ± 3.0) or healthy controls (1.7 ± 1.6), and between PD patients and healthy controls.

Supplementary Table 2 The coefficient of variation of each experiment for α-synuclein SiNaPs and stool samples

	Coefficient of variation [%]	
Experiment	α-synuclein SiNaPs	Stool samples
1	27.22	40.53
2	16.74	25.32
3	17.41	19.34
4	37.19	27.92
5	33.25	22.12
6	25.32	22.11
7	33.05	28.82
8	28.50	25.50
9	36.98	25.31
Mean	28.41	26.33

Supplementary Table 3 LOD values for α-synuclein SiNaPs for each experiment

Experiment	LOD [fM]
1	0.06
2	–
3	0.05
4	0.72
5	0.15
6	0.11
7	0.22
8	0.69
9	0.43
Mean	0.30

LOD, limit of detection

Supplementary Table 4 Number of stool samples above the LOD for each individual experiment

Experiment	Number of samples measured / number of samples above the LOD
1	27 / 27
2	27 / 27
3	24 / 24
4	27 / 27
5	26 / 26
6	26 / 26
7	26 / 26
8	25 / 25
9	9 /9
Sum	**217 / 217**

LOD, limit of detection

Supplementary Table 5 P-values of tests on normal distribution for α-synuclein aggregate concentrations in stool

	PD	iRBD	HC
Shapiro Wilk	0	0	$2.0*10^{-12}$
Lilliefors	$3.5*10^{-39}$	$5.4*10^{-59}$	$5.1*10^{-15}$
Kolmogorov Smirnov	$6.0*10^{-13}$	$1.7*10^{-19}$	$2.5*10^{-5}$
Anderson Darling	$3.7*10^{-54}$	$9.7*10^{-61}$	$1.2*10^{-21}$

PD, Parkinson’s disease; iRBD, isolated rapid eye movement sleep behavior disorder; HC, healthy control

Supplementary Table 6 Spearman coefficient of correlation for α-synuclein aggregate concentrations in stool and age, education, sex, DemTect score, disease duration, MDS-UPDRS III, or questionnaires for PD or iRBD

	All cohorts	PD	iRBD	HC
Age	0.183*	0.125	0.155	0.195
Education	0.041	0.088	0.124	−0.187
Sex	0.008	0.007	−0.074	0.202
DemTect	0.074	0.221*	−0.166	−0.140
Disease duration	−0.050	−0.003	0.069	N/A
MDS-UPDRS III	−0.282*	−0.0004	−0.210	N/A
Screening questionnaire for parkinsonism	−0.154*	0.057	0.031	0.066
RBDSQ	0.188*	0.223*	−0.020	−0.054

PD, Parkinson's disease; iRBD, isolated rapid eye movement sleep behavior disorder; HC, healthy control; MDS-UPDRS, Movement Disorder Society's Unified Parkinson's Disease Rating Scale Part III; N/A, not applicable; RBDSQ, REM sleep behavior disorder screening questionnaire; * $p < 0.05$
