# Supplementary material for: Patients with isolated REM-sleep behavior disorder have elevated levels of alpha-synuclein aggregates in stool
Source: NPJ Parkinsons Dis. 2023 Feb 2;9:14. doi: 10.1038/s41531-023-00458-4 (PMC9895074; doi:10.1038/s41531-023-00458-4)
Supplement: Supplementary file 2 — Reporting Summary [file 41531_2023_458_MOESM2_ESM.pdf]

nature portfolio

Corresponding author(s): Gültekin Tamgüney

Last updated by author(s): Dec 13, 2022

Reporting Summary

Nature Portfolio wishes to improve the reproducibility of the work that we publish. This form provides structure for consistency and transparency in reporting. For further information on Nature Portfolio policies, see our Editorial Policies and the Editorial Policy Checklist.

Statistics

For all statistical analyses, confirm that the following items are present in the figure legend, table legend, main text, or Methods section.

n/a	Confirmed	
☐	☒	The exact sample size (*n*) for each experimental group/condition, given as a discrete number and unit of measurement
☐	☒	A statement on whether measurements were taken from distinct samples or whether the same sample was measured repeatedly
☐	☒	The statistical test(s) used AND whether they are one- or two-sided<br>*Only common tests should be described solely by name; describe more complex techniques in the Methods section.*
☐	☒	A description of all covariates tested
☐	☒	A description of any assumptions or corrections, such as tests of normality and adjustment for multiple comparisons
☐	☒	A full description of the statistical parameters including central tendency (e.g. means) or other basic estimates (e.g. regression coefficient) AND variation (e.g. standard deviation) or associated estimates of uncertainty (e.g. confidence intervals)
☒	☐	For null hypothesis testing, the test statistic (e.g. *F*, *t*, *r*) with confidence intervals, effect sizes, degrees of freedom and *P* value noted<br>*Give P values as exact values whenever suitable.*
☒	☐	For Bayesian analysis, information on the choice of priors and Markov chain Monte Carlo settings
☒	☐	For hierarchical and complex designs, identification of the appropriate level for tests and full reporting of outcomes
☐	☒	Estimates of effect sizes (e.g. Cohen's *d*, Pearson's *r*), indicating how they were calculated

Our web collection on statistics for biologists contains articles on many of the points above.

Software and code

Policy information about availability of computer code

Data collection	InCell Analyzer 6500 7.4
Data analysis	For image data analysis, we used the sFIDAta software application (version 0.61.3), which can be made available upon request. Statistical analyses were performed using SigmaPlot 11.0 , IBM SPSS Statistics 28.0.1.1, and OriginPro 2020 SR1.

For manuscripts utilizing custom algorithms or software that are central to the research but not yet described in published literature, software must be made available to editors and reviewers. We strongly encourage code deposition in a community repository (e.g. GitHub). See the Nature Portfolio guidelines for submitting code & software for further information.

Data

Policy information about availability of data

All manuscripts must include a data availability statement. This statement should provide the following information, where applicable:

- Accession codes, unique identifiers, or web links for publicly available datasets
- A description of any restrictions on data availability
- For clinical datasets or third party data, please ensure that the statement adheres to our policy

The authors confirm that the data supporting the findings of this study are available within the article and its supplementary materials.

Human research participants

Policy information about studies involving human research participants and Sex and Gender in Research.

Reporting on sex and gender	All enrolled subjects completed a basic characteristics questionnaire providing information on various items inkluding sex. There was a gender bias towards men in the PD (69.1%) and more so in the iRBD (86.1%) cohort. In the control group, gender bias was slightly more towards women (60.8%). We could find no correlation of α-synuclein aggregate concentrations and gender.
Population characteristics	All enrolled subjects completed a basic characteristics questionnaire providing information on items such as age, sex, handedness, years of education, and medical history.
Recruitment	PD patients were recruited from patient charts of our outpatient clinic or the ward when patients were admitted to the hospital. IRBD patients were part of an ongoing study recruiting a local prodromal PD cohort in Cologne through newspaper advertisements from the community, followed by a structured telephone interview. Healthy controls were recruited by advertisement, and, in many cases, the caregivers of the PD patients agreed to participate in the study.
Ethics oversight	This study was approved by the Ethics Commission of the Faculty of Medicine of the University of Cologne (19-1644).

Note that full information on the approval of the study protocol must also be provided in the manuscript.

Field-specific reporting

Please select the one below that is the best fit for your research. If you are not sure, read the appropriate sections before making your selection.

☒ Life sciences ☐ Behavioural & social sciences ☐ Ecological, evolutionary & environmental sciences

For a reference copy of the document with all sections, see nature.com/documents/nr-reporting-summary-flat.pdf

Life sciences study design

All studies must disclose on these points even when the disclosure is negative.

Sample size	No sample-size calculation was performed. Samle sizes depended on availability of stool samples of included patients and controls. Sample sizes are sufficient for statistical analysis.
Data exclusions	Healthy controls with an RBDSQ score greater than five were excluded from the analysis.
Replication	Fifty randomly selected stool samples were measured on two different days. Both measurements showed a significant correlation. Additionally, the α-synuclein SiNaPs standard used for calibration was repeatedly measured in nine independent assays, also showing a significant correlation.
Randomization	Randomization is not relevant for this study because groups were pre-defined based on clinical assesment.
Blinding	Researchers were aware of the clinical data at the time of the sFIDA measurement. Blinding was not relevant to this study, since data aquisition could not be influenced and data analysis was performed with the in-house developed sFIDAta software tool to ensure unbiased and automated image data analysis.

Reporting for specific materials, systems and methods

We require information from authors about some types of materials, experimental systems and methods used in many studies. Here, indicate whether each material, system or method listed is relevant to your study. If you are not sure if a list item applies to your research, read the appropriate section before selecting a response.

Materials & experimental systems

n/a	Involved in the study	
☐	☒	Antibodies
☒	☐	Eukaryotic cell lines
☒	☐	Palaeontology and archaeology
☒	☐	Animals and other organisms
☒	☐	Clinical data
☒	☐	Dual use research of concern

Methods

n/a	Involved in the study	
☒	☐	ChIP-seq
☒	☐	Flow cytometry
☒	☐	MRI-based neuroimaging

Antibodies

Antibodies used	α-synuclein antibody (211): Santa Cruz Biotechnology Inc. (Dallas, USA); catalog number: sc-12767, lot number: #A2022
Validation	Santa Cruz Biotechnology antibodies have over 360,000 research citations. Anti-α-synuclein Antibody (211) has 211 citations in a variety of scientific publications. Primary antibodies like Anti-α-synuclein Antibody (211) for mammalian target proteins are recommended for the detection of a range of mammalian species, primarily of mouse, rat and human species.
